# Acute hypoxia induced dysregulation of clock-controlled ovary functions

**DOI:** 10.3389/fphys.2022.1024038

**Published:** 2022-12-23

**Authors:** Mengnan Ding, Yarong Lu, Xin Huang, Chen Xing, Shaojun Hou, Dongxue Wang, Yifan Zhang, Wei Wang, Chongchong Zhang, Min Zhang, Fanfei Meng, Kun Liu, Guangchao Liu, Jincheng Zhao, Lun Song

**Affiliations:** ^1^ Beijing Institute of Basic Medical Sciences, Beijing, China; ^2^ Henan University Joint National Laboratory for Antibody Drug Engineering, Kaifeng, China; ^3^ Anhui Medical University, Hefei, China; ^4^ School of Pharmacy, Jiamus University, Jiamusi, China; ^5^ College of Life Science, Henan Normal University, Xinxiang, China

**Keywords:** hypobaric hypoxia, circadian clock, reproductive disorder, ovulation, high altitude

## Abstract

High altitudes or exposure to hypoxia leads to female reproductive disorders. Circadian clocks are intrinsic time-tracking systems that enable organisms to adapt to the Earth’s 24-h light/dark cycle, which can be entrained by other environmental stimuli to regulate physiological and pathological responses. In this study, we focused on whether ovarian circadian clock proteins were involved in regulating female reproductive dysfunction under hypoxic conditions. Hypobaric hypoxia was found to induce a significantly prolonged estrous cycle in female mice, accompanied by follicular atresia, pituitary/ovarian hormone synthesis disorder, and decreased LHCGR expression in the ovaries. Under the same conditions, the levels of the ovarian circadian clock proteins, CLOCK and BMAL1, were suppressed, whereas E4BP4 levels were upregulated. Results from granulosa cells (GCs) further demonstrated that CLOCK: BMAL1 and E4BP4 function as transcriptional activators and repressors of LHCGR in ovarian GCs, respectively, whose responses were mediated by HIF1ɑ-dependent (E4BP4 upregulation) and ɑ-independent (CLOCK and BMAL1 downregulation) manners. The LHCGR agonist was shown to efficiently recover the impairment of ovulation-related gene (*EREG* and *PGR*) expression in GCs induced by hypoxia. We conclude that hypoxia exposure causes dysregulation of ovarian circadian clock protein (CLOCK, BMAL1, and E4BP4) expression, which mediates female reproductive dysfunction by impairing LHCGR-dependent signaling events. Adjusting the timing system or recovering the LHCGR level in the ovaries may be helpful in overcoming female reproductive disorders occurring in the highlands.

## 1 Introduction

Continuous economic development has resulted in the number of women working and traveling in high-altitude areas (especially female soldiers) increasing annually. Multiple epidemiological studies have shown that short- and long-term stays in the highlands lead to significant menstrual cycle disorder and hormone level fluctuation in women, with some exhibiting fetal growth restriction and impaired fertility (West 2017; Shaw et al., 2018; Lorca et al., 2019). Female reproductive function is controlled by the hypothalamus–pituitary–ovarian (HPO) axis. Sympathetic signals and gonadotropins released by the pituitary gland, such as follicle-stimulating hormone (FSH) and luteinizing hormone (LH), can regulate reproductive events that occur in the ovary, uterus, and oviduct, including follicular growth and differentiation, steroid hormone synthesis and secretion, ovulation, implantation, and parturition (Mikhael et al., 2019). Dysregulation of the HPO axis and correlated signal events results in menstrual cycle disorders and decreased fertility (Mikhael et al., 2019).

High-altitude exposure leads to hypobaric hypoxia, which could induce physiological dysfunction and pathological responses, such as acute mountain sickness (AMS), high-altitude cerebral edema (HACE), and high-altitude pulmonary edema (HAPE) (Basnyat and Murdoch 2003). Previous studies have also shown that hypobaric hypoxia affects female reproductive health. For instance, it seemed that the high-altitude hypoxia was positively related with retarded menarche, low fertility, and high infant mortality (Shaw et al., 2018). Consistent with the results of human-based studies, data from the corresponding animal experiments also indicate clear reproductive dysfunction in female mice, rats, and sheep exposed to hypobaric hypoxia, including irregular estrous cycle, abnormal hormone secretion, ovarian morphological changes, luteal dysfunction, and reductions in birth weight (Parraguez et al., 2013; Määttä et al., 2018; Shaw et al., 2020). The development of hypobaric hypoxia-related pathologies was thought to be related to oxidative stress inflammation and fibrosis (Pena et al., 2020; Pena et al., 2022). Apart from these well-accepted causative factors, circadian rhythm disturbance may also participate in hypobaric hypoxia-evoked dysfunctions. Indeed, a significant increase in nocturnal melatonin release was observed after ascent to high altitude (Frisch et al., 2004). However, the exact role and the functional mechanism of the circadian clock in hypobaric hypoxia-related fertility in female reproductive deficiency remain unclear.

Circadian clocks are intrinsic time-tracking systems that allow organisms to display physiological and behavioral oscillations with a period of about 24 h (Takahashi 2017). The central clock, located in the suprachiasmatic nucleus (SCN) of the hypothalamus, and the peripheral clocks throughout the body constitute the mammalian circadian system, which influences most physiological processes, such as sleep, metabolism, fertility, and cognition processes (Hastings et al., 2018). Among them, the effects of circadian clocks on fertility have drawn increasing attention, especially in the context of global fertility decline. The circadian clock was reported to be involved in follicle development and reproductive hormone homeostasis (Rahman et al., 2019; Shao et al., 2021), while circadian clock mutation perturbed the estrous cyclicity as well as the maintenance of pregnancy (Miller et al., 2004). Specific deletion of *Bmal1* in ovarian theca cells impaired ovulation in female mice (Mereness et al., 2016). An aberrant circadian clock gene expression profile was also observed in polycystic ovary syndrome (PCOS) patients (Johnson et al., 2022), strongly indicating the significant role of the circadian system in female fertility.

Circadian rhythms are encoded by a transcriptional–translational feedback loop composed of transcription activators (CLOCK and BMAL1) that induce the expression of transcriptional repressors (PER1-3 and CRY1-2) by binding to E-box motifs within their promoter regions. The accumulation of PER and CRY proteins results in feedback repression of CLOCK:BMAL1 activity, terminating PER and CRY transcription. PER:CRY complexes are then degraded, inhibiting their negative feedback action on the CLOCK:BMAL1 heterodimer. Additionally, CLOCK:BMAL1 also induces the expression of RORα and REV-ERBα, which activate or repress BMAL1 transcription, respectively, by binding to the REV-ERB/ROR response element (RRE) within its promoter. Furthermore, E4BP4 and three PAR bZip family members (DBP, TEF, and HLF) also form a regulatory network of rhythmic gene expression by recruitment to the D-box element. Despite the auto-regulatory and self-sustained properties, the clock system could also be entrained by external environmental cues, such as feeding schedules, exercise, and stress (Patke et al., 2020). Hypoxia is among the stress factors that could influence the circadian clock. Intermittent hypoxia was reported to alter the clock gene expression in the mouse brain and liver (Koritala et al., 2021). In addition to that, hypoxia also induced inter-tissue circadian clock misalignment, which was regarded as a potential contributor to the pathophysiology of diseases that involve hypoxia (Manella et al., 2020). Mechanistically, it was hypothesized that hypoxia affects the circadian system by the transactivity of hypoxia-inducible factors (HIFs) at the circadian clock genes. Indeed, HIF-1α was found to be able to bind directly to PER2 promoter and regulate its expression (Wu et al., 2017).

Physiologically, circadian clocks are critical for maintaining homeostasis in the female HPO axis, including gonadotropin secretion, follicular maturation, ovarian steroid hormone production, ovulation, uterine implantation, and parturition. The disruption of the molecular clock in the HPO axis or reduced synchrony among these oscillators may contribute to impaired fertility (Sellix 2015; Sen and Sellix 2016; Sen and Hoffmann 2020; Brzezinski et al., 2021; Shao et al., 2021). Sleep disorder frequently occurs as a stress response to the highlands, which results in disturbances in circadian rhythms and a variety of physiological abnormalities (Windsor and Rodway 2012; Bloch et al., 2015). Therefore, in the current study, we investigated whether hypobaric hypoxic exposure induced disorder of the circadian clock protein expression in the ovaries and its contribution to female reproductive dysfunction occurring in the highlands.

## 2 Materials and methods

### 2.1 Animals and treatments

Eight-week-old non-mated female C57BL/6 mice were obtained from Charles River Laboratory Animal Technology Co. (Beijing, China). The animals were housed in individual cages in a climate-controlled room under 12-h/12-h light/dark cycles and 50% relative humidity for 1 week. Food and water were provided *ad libitum*. The female mice were all in the estrus stage when they were exposed to hypoxic conditions. All animal experiments were approved by the Ethics Committee of the Beijing Institute of Basic Medical Sciences and carried out in accordance with the approved guidelines.

The effect of hypobaric hypoxia on serum hormone levels was evaluated on female mice raised under normoxic (21% O_2_) or hypobaric hypoxic conditions (simulated altitude of 6,000 m, 11.4% O_2_, and 10.7-kPa O_2_ pressure) at ZT0 (7:00 AM). The mice were euthanized every 4 h at ZT4, ZT8, ZT12, ZT16, ZT20, and ZT24 (*n* = 6), and the serum was collected for subsequent examinations.

The effect of hypobaric hypoxia on the expression of clock proteins in the ovary was evaluated on female mice raised under normoxic or hypobaric hypoxic conditions as described previously at ZT0 (*n* = 6). The mice were euthanized after 48 h, and ovarian tissues of metestrus female mice were collected for subsequent examination.

### 2.2 Estrous cycle determination

The estrous cycle of the mice was analyzed using a previously published protocol (Ma et al., 2020). A vaginal smear from each mouse was stained with a methylene blue solution to determine the different phases. The proestrous phase is characterized by numerous nucleated epithelial cells; the estrous phase is characterized by non-nucleated cornified cells; the metestrous phase is characterized by nucleated epithelial cells, non-nucleated cornified cells, and leukocytes; and the diestrous phase is characterized by many leukocytes. Mice with a normal progressive cycle (total duration of 4–5 days) were considered to have a regular estrous cycle and were subjected to hypobaric hypoxic conditions.

### 2.3 Ovarian follicular histological analysis

The ovaries of control and hypoxia-treated mice were collected and subjected to HE staining, as described previously (Li et al., 2016). Follicle categorization was performed as follows: primordial follicles were classified as oocytes surrounded by a single layer of flattened follicular cells; primary follicles were classified as flattened follicle cells that became squamous or cuboidal (so-called granulosa cells); and secondary follicles were classified on the basis of a visible follicular antrum. In follicles with an enlarged antrum, the cumulus oophorous was diminished, leaving the free-floating oocyte surrounded by two or three layers of granulosa cells (GCs). After this stage, the follicles bulged outward from the ovary and were classified as mature. Typical interstitial glands and follicles with shrunken oocytes or GCs that had begun to disaggregate were categorized as atretic follicles. The total number of follicles and atretic follicles per ovary was determined. The percentage of atretic follicles of the total follicles was calculated.

### 2.4 Cell culture and treatment

Ovaries were harvested from eight-week-old C57BL/6 female mice (*n* = 10). Follicles were punctured using a 1-ml syringe needle. The mixture was filtered using a cell strainer (200 mesh). The cumulus–oocyte complexes and ovarian tissues were discarded. Thereafter, GCs were pelleted by centrifugation (10 min at 1,000 × *g*) and cultured in six-well plates with DMEM containing 10% fetal bovine serum (Biological Industries, 04-001-1ACS), 100 U/mL penicillin, 50 mg/ml streptomycin, and recombinant follicle-stimulating hormone (FSH) (PRS-HOR-253). The GCs were incubated in a humidified atmosphere containing 5% CO_2_ at 37 °C for 4 days. KK1 mouse ovarian GCs were provided by Dr. Wentao Xu (China Agricultural University, China). The cells were maintained in DMEM with 10% fetal bovine serum supplemented with penicillin and streptomycin. GCs and KK1 cells were cultured under normoxic (21% O_2_) or hypoxic (1% O_2_) conditions, and then cell lysates were prepared for subsequent examinations. As for the treatment of KK1 cell with CG, KK1 cells were treated with CG (PRS-HOR-250, ProSpec Ltd) at a concentration of 1 IU for 6 h.

### 2.5 TUNEL assay

*In situ* detection of apoptosis in the ovaries was performed using a TUNEL apoptosis assay kit (C1086, Beyotime Biotechnology), following the manufacturer’s guidelines. Stained paraffin sections were subsequently counter-stained with DAPI (C0060, Solarbio), followed by visualization with microscopy (IX73, Olympus).

### 2.6 Hormone level assay

At the corresponding time point, blood was sampled from the mice eye sockets when the control mice were in the estrus stage; GC culture supernatants were harvested. The collected sample was stored at −80 °C until assayed. The levels of progesterone (P_4_, CEA459Ge), 17B-estradiol (E_2_, CEA461Ge), LH (CEA441Mu), FSH (SEA830Mu), and corticosterone (CEA540Ge) were measured using enzyme-linked immunosorbent assay (ELISA) kits purchased from Cloud-Clone Corp. (Wuhan, China), according to the manufacturer’s protocols.

### 2.7 siRNA transfection

siRNA transfection was conducted using Lipofectamine ^®^RNAiMAX (13778-150, Invitrogen), according to the manufacturer’s instructions. Sequences of siRNA targeting HIF-1α, BMAL1, CLOCK, and E4BP4 are available upon request.

### 2.8 Protein extraction and Western blot assay

Ovaries were homogenized in lysis buffer to obtain protein lysates, and protein concentrations were determined using a bicinchoninic acid assay (Thermo Fisher Scientific, United States). Equal amounts of protein (120 μg) were separated by electrophoresis using 8–10% sodium dodecyl sulfate polyacrylamide gels (SDS-PAGE). The separated proteins were transferred to polyvinylidene fluoride (PVDF) membranes. The membranes were incubated in 5% non-fat milk diluent for 1 h and then incubated overnight with primary antibodies targeting LHCGR (1:1,000, Proteintech, 19968-1-AP), FSHR (1:1,000, Proteintech, 22665-1-AP), BMAL1 (1:1,000, Proteintech, 14268-1-AP), CRY1 (1:1,000, Proteintech, 13474-1-AP), β-actin (1:5,000, Proteintech, 20536-1-AP), CLOCK (1:200, Santa Cruz Biotechnology, sc-271603), E4BP4 (1:200, Santa Cruz Biotechnology, sc-74415), PER2 (1:1,000, GeneTex, GTX129688), RORα (1:1,000, Novus, NBP2-24519), REV-ERBα (1:1,000, Abcam, ab174309), and HIF-1α (1:1,000, Cell Signaling Technology, 36169) at 4 °C. The following day, the membranes were washed and incubated with secondary antibodies conjugated with horseradish peroxidase (HRP) for 1 h at room temperature. An ECL detection reagent (Kangwei Century Biotechnology, China) was used to detect the protein band signals. Data were obtained from independent experiments performed in triplicate.

### 2.9 RNA isolation and RT-PCR assay

Total RNA was extracted using TRIzol reagent (Sigma-Aldrich, T9424), and the cDNA products were synthesized using the Thermo Script^TM^ RT-PCR system (Thermo Fisher Scientific, M1631). The following oligonucleotides were synthesized and used to amplify target cDNAs: Lhcgr: 5′-cca​cgc​tga​ccc​tag​c-3′ (forward) and 5′-tcc​aga​gtg​atg​aag​cgt​ctc​g-3′ (reverse); Fshr: 5′-ctt​tgc​cat​ttc​cgc​ctc​cc-3′ (forward) and ‘5′-tgc​cag​ctc​ttc​cat​agc​cc-3′ (reverse); Ptgs2: 5′-tac​cgc​aaa​cgc​ttc​tcc​ct-3′ (forward) and 5′-cac​ttc​gcc​tcc​aaa​ggt​gc-3′ (reverse); Areg: 5′-cct​tcg​ctc​cct​tct​gac​cct-3′ (forward) and 5′-gcc​tga​gcc​taa​gac​cag​ca-3′ (reverse); Ereg: 5′-ggc​agc​tat​cta​gag​agc​ca -3′ (forward) and ‘5′-cca​gaa​tgc​ctt​gtg​cgc​tg-3′ (reverse); Pgr: 5′-aaa​act​gcc​cag​cat​gtc​gt -3′ (forward) and ‘5′-aag​agc​tgg​aag​tgt​cag​gc-3′ (reverse); *E4BP4* : 5′-gga​gca​gaa​cca​cga​taa​ccc-3′ (forward) and 5′-ccc​atg​ctc​cct​tcg​ttc​ag-3′ (reverse); *BMAL1*: 5′- atc​ttc​ctc​gga​cac​tgc​ac-3′ (forward) and 5′- acc​cgt​att​tcc​ccg​ttc​ac-3′ (reverse); and *CLOCK*: 5′- agt​tag​ggc​tga​aag​acg​gc-3′ (forward) and 5′- aca​tgc​ctt​gtg​gga​ttg​gt-3′ (reverse). The primers used to amplify GAPDH cDNA were 5′-agc​tat​gcg​ctg​cct​gac​gg-3' (forward) and 5′-gcagctcagtaacagtccgc-3′(reverse).

### 2.10 Luciferase assay

The mouse luteinizing hormone receptor (LHCGR) promoter sequence (1,000 bp before the transcription start site) was obtained by PCR and then inserted into the pGL3 basic vector to construct the LHCGR-pro-Luc reporter plasmid. The luciferase reporter plasmid was transfected into KK1 cells, in combination with siRNA specifically targeting BMAL1, CLOCK, E4BP4, HIF1ɑ, or their control siRNAs. Cells were collected 36 h after transfection, and luciferase activity was tested using a luciferase assay system (Promega, USA).

### 2.11 ChIP assay

The ChIP assay was performed using the SimpleChIP enzymatic chromatin IP kit (Cell Signaling Technology, 9002) according to the manufacturer’s protocol. The following primers were used to amplify the regions covering the putative E-box or D-box within the mouse LHCGR or HIF1ɑ-responsive element (HRE) within the mouse E4BP4 promoter. E-box1 within mouse LHCGR promoter (−788 to −784): 5′-cct​ggg​gct​gct​gta​aga​tc-3′ (forward) and 5′-agg​aac​ata​ata​ccc​aag​gt-3′ (reverse); E-box2 within mouse LHCGR promoter (−854 to −850): 5′-cga​tgc​cta​atg​aac​acc​gta​gag-3′ (forward) and 5′-gtg​gat​gga​gga​gtg​ctt​ttt​gac-3′ (reverse); D-box1 within mouse LHCGR promoter (−198 to −192): 5′-gac​cat​ggc​aga​gca​gag​ttc​aaa-3′ (forward) and 5′-gtc​cca​ggt​caa​gga​gaa​cag​g-3' (reverse); D-box2 within mouse LHCGR promoter (−425 to −419): 5′-gga​cag​acc​caa​ata​gct​atc​tg-3′ (forward) and 5′-act​ttg​gct​ctt​ccg​atg​tgg​ata-3′ (reverse); HRE1 within mouse E4BP4 promoter: (−267 to −263): 5′-tgc​tgg​cac​cac​gga​gcg​a-3′ (forward) and 5′-agc​tgc​tac​ccg​ctg​tcc​gt-3′ (reverse); and HRE2 within mouse E4BP4 promoter (−832 to −828): 5′-aca​tgc​cac​cag​atg​ctc​tgt-3′ (forward) and 5′-cgc​ttt​cgg​tgc​tcg​ggc​t-3′ (reverse).

### 2.12 Immunofluorescence assay

KK1 cells transfected with siRNA targeting BMAL1, CLOCK, or E4BP4 were subjected to immunofluorescence assay to determine the influences of BMAL1, CLOCK, and E4BP4 on the expression of LHCGR. Briefly, the transfected cells were fixed in 4% formaldehyde and permeabilized with 0.3% Triton X-100. Cells were then blocked with 5% goat serum in PBS-T, followed by overnight incubation with anti-LHCGR antibody (1:100). On the second day, cells were washed and incubated with Alexa Fluor^®^ 488 conjugate (A11008, Invitrogen). Cells were then counter-stained with DAPI at room temperature. The stained cells were visualized using microscopy (IX73, Olympus).

### 2.13 Statistical analysis

Data from the hypoxia-treated and control groups were compared using a *t*-test and are presented as mean ± standard deviation (SD). Data were analyzed using SPSS 19.0 and GraphPad Prism 6 software. Statistical significance was set at *p* < 0.05.

## 3 Results

### 3.1 Estrous cycle disorder and follicular atresia

**FIGURE 1:**
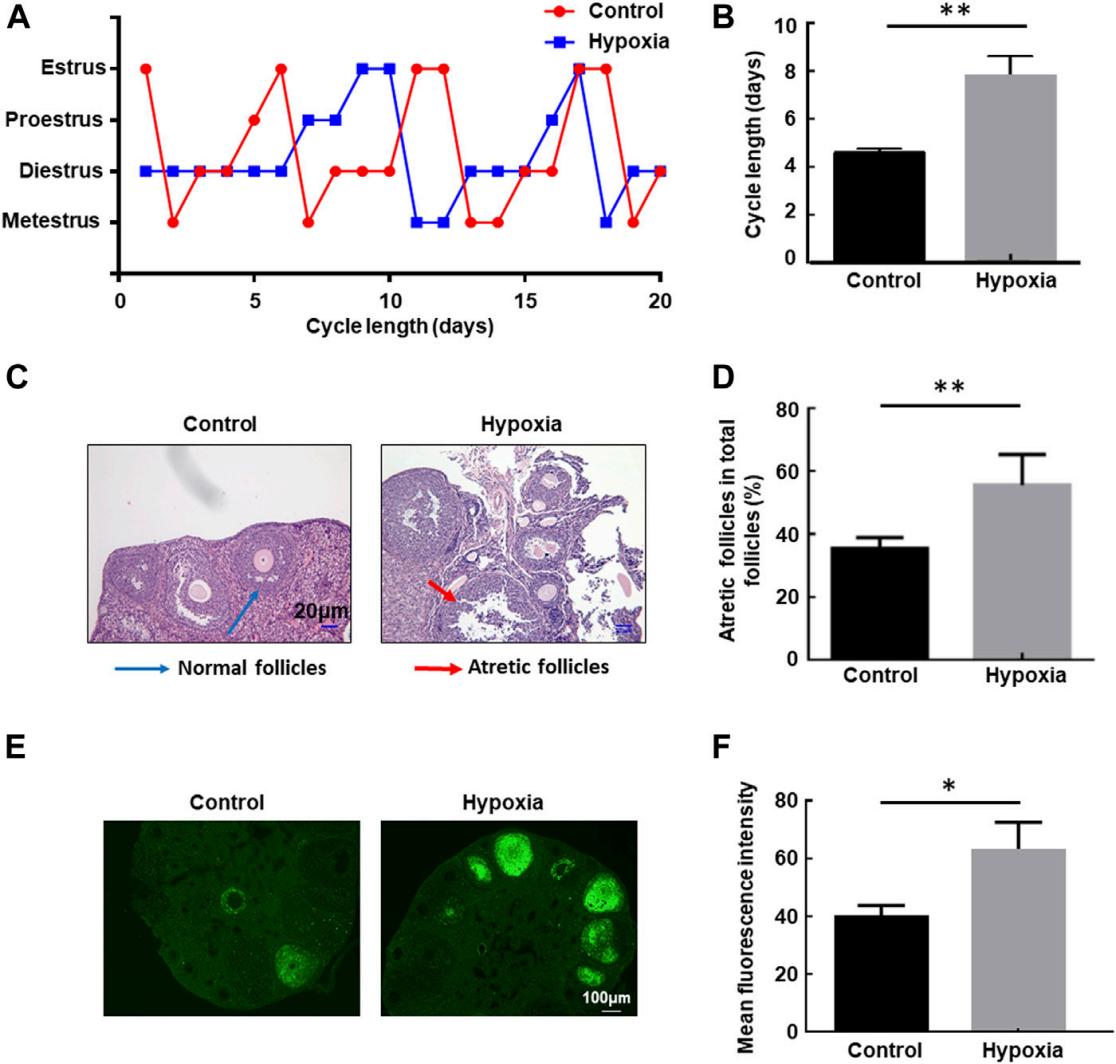
Hypobaric hypoxia-induced estrous cycle disorder and follicular atresia in female mice. **(A–B)** Eight-week-old unmated female mice were left untreated or exposed to hypobaric hypoxic conditions that simulated 6000-m altitude for 48 h, and then the estrous cycle of each group mice was examined in the following three weeks. The distribution of estrous cycles of representative untreated and hypoxia-treated mice is shown in **(A)**. The average estrous cycle length of mice from each group is shown in **(B)** (*n* = 20, **p < 0.01). **(C–D)** Female mice were raised under normoxic or hypobaric hypoxic conditions at ZT0. Mice were euthanized after 48 h, and the ovaries were collected and subjected to HE staining. Follicle categorization was performed, and the percentage of atretic follicles of the total follicles was calculated. (*n* = 6, **p < 0.01). **(E)** TUNEL assay of apoptotic cells in the ovaries after hypoxic exposure. **(F)** Quantification of the TUNEL assay (*n* = 3, *p < 0.05).

The health risk of hypobaric hypoxia on the female reproductive system was evaluated using eight-week-old unmated female mice. These mice were untreated or exposed to hypobaric hypoxic conditions that simulated 6000-m altitude for 48 h, and then the estrous cycle of each group of mice was examined in the following 3 weeks. Hypobaric hypoxia significantly prolonged the estrous cycle of female mice (Figure 1A). The cycle length increased from approximately 4.5 days under normal conditions to approximately 8 days after exposure to acute hypoxia (*p* < 0.01, Figure 1B). These results indicated that hypobaric hypoxia exposure induces estrous cycle disorders in female mice.

Next, we examined the follicular morphology before and after exposure to hypoxia. The percentage of atretic follicles (shrunken oocytes and disaggregated GCs) in the ovaries of the stressed mice increased significantly compared with the untreated mice (Figures 1C, D), indicating that hypobaric hypoxia induces defects in follicular development in female mice. Furthermore, a TUNEL assay was performed to detect apoptosis in the ovaries. A significantly increased immunofluorescence signal for TUNEL-positive cells in the ovaries of mice exposed to hypoxia was observed compared with that of control mice under the same conditions (Figures 1E, F), providing further evidence for follicular atresia in the ovary, induced by hypoxia.

### 3.2 Impairment of LH/LH receptor (LHCGR) pathway activation

**FIGURE 2:**
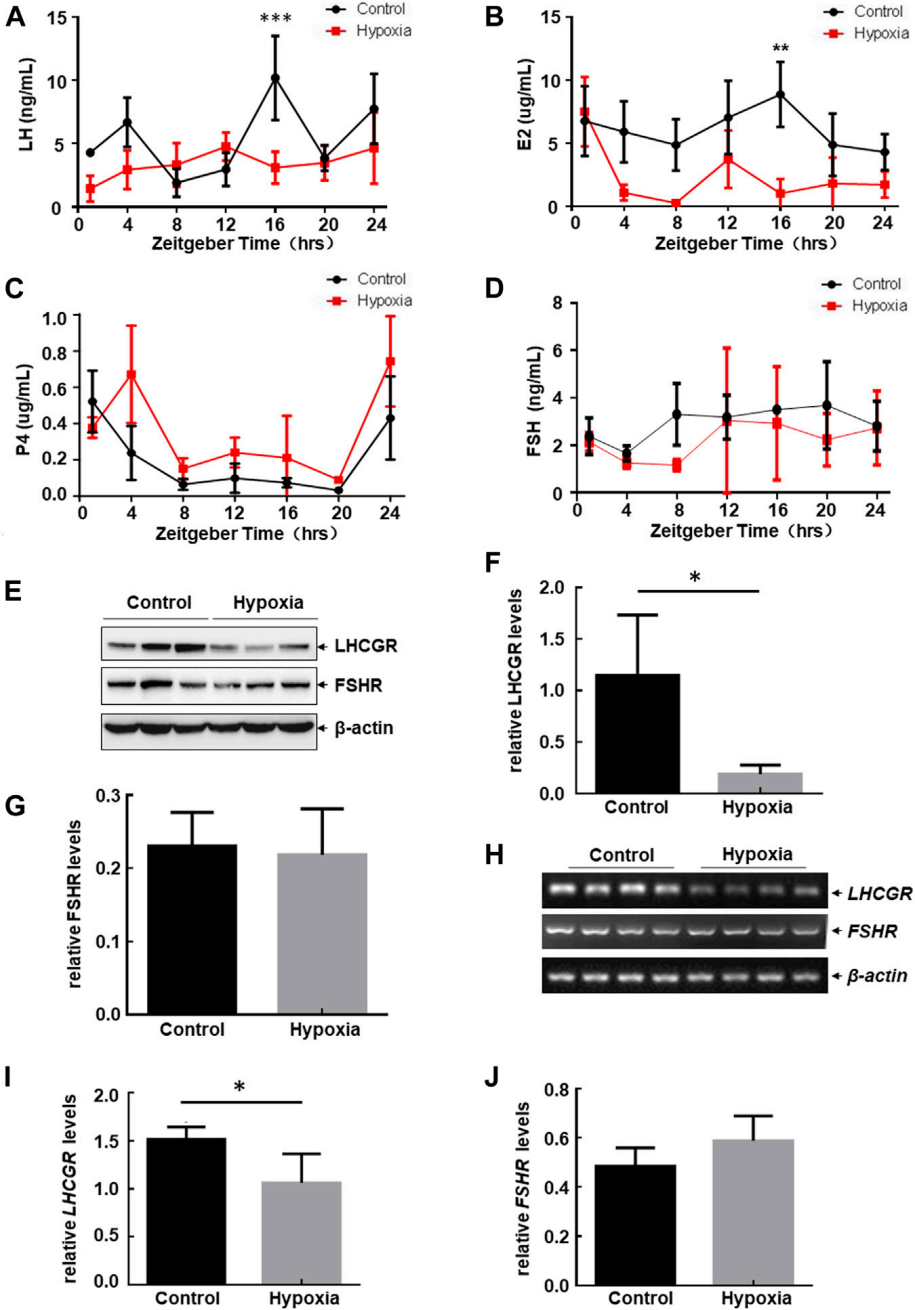
Hypobaric hypoxia induced the impairment of LH-LHCGR pathway activation in the ovaries of female mice. **(A–D)** Female mice were exposed to hypobaric hypoxia. The mice were euthanized every 4 h. Serum levels of sex hormones were analyzed by ELISA (*n* = 6, ***p < 0.0001; **p < 0.01). **(E–J)** Female mice were raised under normoxic or hypobaric hypoxic conditions at ZT0. Ovaries were collected 48 h after hypoxic exposure and subjected to Western blot (E) or RT-PCR assays (H). The quantitative results of each target protein (relative to β-actin) are shown **(F–G, I–J)** (*n* = 6–8, *p < 0.05).

Serum hormone and hormone receptor levels expressed in the ovary play important roles in regulating follicular development and the estrous cycle in female mice (Mikhaelet al. 2019). Therefore, to determine the mechanism involved in estrous cycle disorder and follicular atresia under hypoxic exposure, we determined the serum levels of hormones secreted by the pituitary gland and ovary with and without hypoxia exposure at different ZTs. Rhythmic expression of LH, E_2_, and P_4_ was observed in the serum within 24 h (Figures 2A–C), while the serum level of FSH remained stable under the same steady-state conditions (Figure 2D). After hypoxic exposure, the 24-h oscillatory expression of serum LH almost disappeared, with a significant decrease in expression observed at ZT16 (Figure 2A). E_2_ levels were also downregulated at ZT16 (Figure 2B). However, FSH and P_4_ levels showed no change before and after hypoxic exposure (Figures 2C, D). Together, these results indicate that hypobaric hypoxia induces hormone disorders in the pituitary gland (LH) and ovary (E_2_) in female mice.

Because E_2_ promotes the secretion of LH through positive feedback regulation, thereby promoting the growth and maturation of follicles (Mikhael et al., 2019), and the serum level of LH changes most dramatically after hypoxic stress, we next focused on investigating the possible role of the LH-dependent pathway in hypoxia-induced female reproductive disorder. Here, we found that LH receptor (LHCGR) expression in the ovary decreased significantly after hypoxia exposure (Figures 2E, F), whereas FSH receptor (FSHR) levels did not change under the same conditions (Figures 2E, G). These results indicate that the impairment of ovarian LHCGR expression is also a major response to hypoxic exposure in female mice.

Furthermore, we found that mRNA levels of ovarian LHCGR were also downregulated after hypoxia exposure, while FSHR mRNA levels remained unchanged under the same stress conditions (Figures 2H–J). This was consistent with the Western blot assay results. These data indicated that hypoxia-induced LHCGR reduction resulted from the transcriptional suppression of LHCGR mRNA. After obtaining these results, we propose that high altitudes lead to loss of rhythmic LH synthesis and reduction of ovarian LHCGR expression which will cause the ovary to respond to LH stimulation abnormally. This may be critical for inducing follicular atresia and prolonged estrous cycles in female mice. Therefore, signaling events leading to ovarian LHCGR suppression under hypoxic conditions were evaluated.

### 3.3 Circadian clock protein expression in the ovary

**FIGURE 3:**
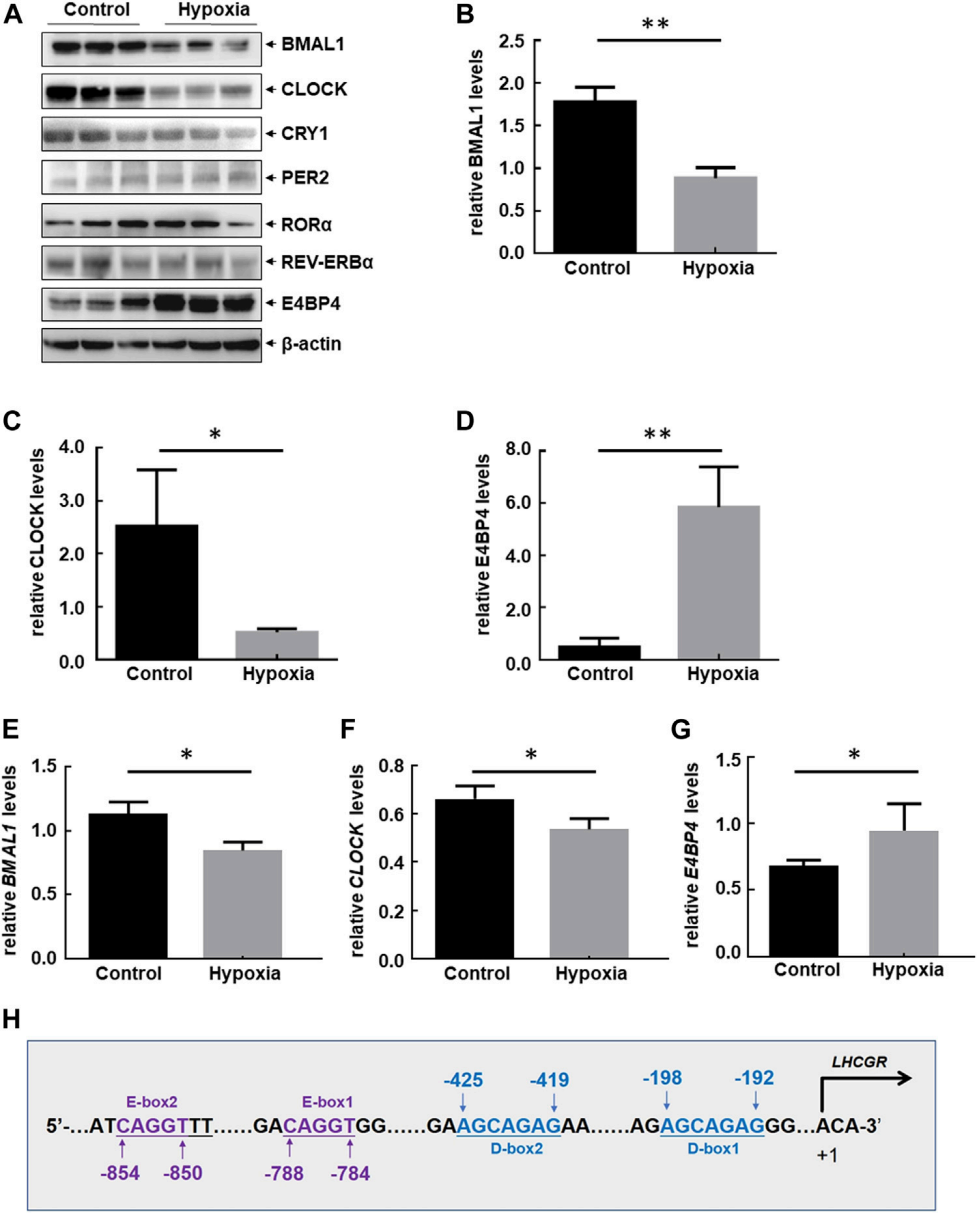
Hypobaric hypoxia-induced disorder of circadian clock protein expression in the ovaries of female mice. **(A–D)** Female mice were treated as shown in Figure 1A. Ovaries were collected 48 h after hypoxic exposure, and the expression levels of circadian clock proteins were detected. The Western blot quantitative results of CLOCK, BMAL1, and E4BP4 (relative to β-actin) are shown **(B–D)** (*n* = 6, **p < 0.01; *p < 0.05). The RT-PCR results of BMAL1, CLOCK, and E4BP4 (relative to *β-actin*) are shown **(E–G)** (*n* = 3, *p < 0.05). **(H)** Potential CLOCK:BMAL1- and E4BP4-binding sites (E-box and D-box) were identified within mouse LHCGR promoter regions.

Circadian clocks are critical for maintaining homeostasis in the female reproductive system (Sellix 2015; Sen and Sellix 2016; Sen and Hoffmann 2020; Brzezinski et al., 2021; Shao et al., 2021). Sleep disorder frequently occurs as a stress response to the highlands, which results in disturbance of the circadian rhythm (Windsor and Rodway 2012; Bloch et al., 2015). Therefore, dysregulation of clock-controlled output signals was investigated for its role in regulating the impairment of ovarian LHCGR expression in response to hypoxia exposure. Levels of circadian clock proteins in the ovary were measured before and after 48 h of hypoxia treatment. A significant reduction in CLOCK and BMAL1 protein accumulation and an increase in E4BP4 protein accumulation in the ovaries of hypoxia-treated mice were observed, while other ovarian clock protein (PER2, CRY1, RORɑ, and REV-ERBɑ) levels did not change compared with those of the control samples (Figures 3A–D). The suppression of CLOCK and BMAL1 and the upregulation of E4BP4 were further confirmed by RT-PCR at the mRNA level (Figures 3E–G). Cis-acting elements within the mouse LHCGR promoter were analyzed, and DNA sequences matching the binding sites for the CLOCK: BMAL1 heterodimer (E-box) and E4BP4 (D-box) were identified (Figure 3H). Therefore, we speculate that CLOCK, BMAL1, and E4BP4 may play key roles in mediating hypoxia-induced LHCGR reduction and reproductive dysfunction in female mice.

### 3.4 LHCGR transcription in granulosa cells

**FIGURE 4:**
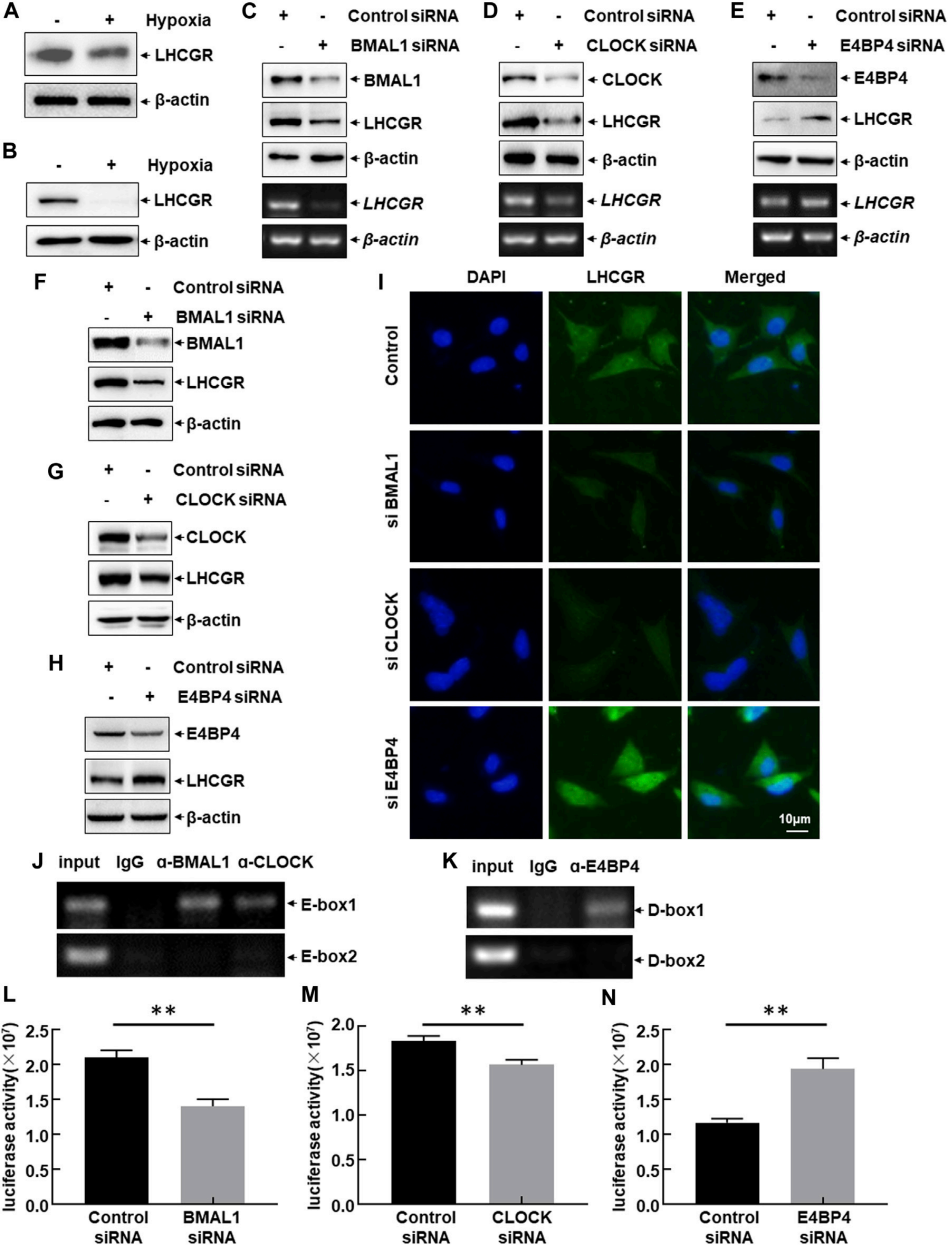
CLOCK, BMAL1, and E4BP4 were involved in LHCGR transcription in the granulosa cells. **(A)** Primary granulosa cells (GCs) were prepared and cultured under normoxic (21% O_2_) or hypoxic (1% O_2_) conditions for 6 h. Cells were then collected to detect LHCGR expression. **(B)** KK1 cells cultured under the same conditions and LHCGR levels detected. **(C–E)** Primary GCs were transfected with CLOCK, BMAL1, E4BP4 siRNA, or control siRNA. Changes in LHCGR mRNA and protein levels were detected. KK1 cells were transfected as described for the primary GCs, and LHCGR levels were detected using Western blot **(F–H)** and *in situ* immunofluorescence staining **(I)**. **(J–K)** ChIP assays were performed in KK1 cells to detect the binding site of CLOCK: BMAL1 heterodimer and E4BP4 within the mouse LHCGR promoter region. **(L–N)** KK1 cells were transfected with the LHCGR promoter-driven luciferase reporter in combination with siRNA targeting CLOCK, BMAL1 or E4BP4 or control siRNAs. Luciferase activity was detected at 36 h after transfection (**p < 0.01).

LHCGR is predominantly expressed in GCs, theca cells (TCs), and luteal cells. The responsiveness of LHCGR expressed in GCs to LH is a key factor in controlling follicular development and ovulation (Troppmann et al., 2013; Mikhael et al., 2019). Therefore, to investigate the intracellular signaling events leading to hypoxia-induced LHCGR suppression, primary GCs were isolated from female mice and then cultured under normoxic (21% O_2_) or hypoxic (1% O_2_) conditions. After 6 h, a decrease in LHCGR expression was observed in hypoxia-treated GCs (Figure 4A). Similar results were obtained in the mouse GC line (KK1) after exposure to hypoxia (Figure 4B). These results suggest that GCs are hypoxia-sensitive ovarian components, and the decreased expression of LHCGR on their surface may play an important role in mediating hypoxia-induced ovarian dysfunction.

The role of CLOCK, BMAL1, and E4BP4 in the suppression of LHCGR expression in granulosa cells after exposure to hypoxia was determined by separately transfecting CLOCK, BMAL1, or E4BP4 siRNA into primary GCs. The expression level of LHCGR was detected. Blocking CLOCK and BMAL1 expression significantly inhibited LHCGR transcription and protein synthesis, whereas knockdown of E4BP4 expression showed the opposite effect (Figures 4C–E). Similar results were observed when the same siRNAs were transfected into KK1 cells, as evidenced by the Western blot results (Figures 4F–H) and the immunofluorescence staining (Figure 4I). These results suggest that CLOCK:BMAL1 and E4BP4 regulate LHCGR expression in GCs as transcriptional activators and repressors, respectively.

The effect of circadian clock proteins on LHCGR transcription was analyzed by performing ChIP assays using specific antibodies against CLOCK, BMAL1, E4BP4, or normal serum. Among the CLOCK:BMAL1 (E-box)- and E4BP4 (D-box)-binding motifs within the mouse LHCGR promoter, the CLOCK:BMAL1 heterodimer was recruited to the proximal site (−788 to −784 bp) (Figure 4J). Additionally, a strong association between E4BP4 and the proximal chromatin region containing the D-box (−198 to −192 bp) was also observed (Figure 4K). No signal was observed in the control immunoglobulin G-immunoprecipitated samples, supporting the specific binding of CLOCK, BMAL1, and E4BP4 to the LHCGR promoter (Figures 4J,K). These results indicate that circadian clock proteins regulate the transcription of LHCGR by recruiting DNA-responsive elements within the LHCGR promoter.

These results were confirmed by performing luciferase assays using the constructed luciferase reporter driven by the mouse LHCGR promoter. Blocking CLOCK and BMAL1 expression significantly inhibited LHCGR promoter-driven luciferase activity, whereas knockdown of E4BP4 expression showed the opposite effect (Figures 4L–N). These results indicate that exposure to hypoxia induces a decrease in CLOCK and BMAL1 expression and an increase in E4BP4 expression, which can synergistically downregulate LHCGR transcription in GCs.

### 3.5 LHCGR reduction by HIF1ɑ

**FIGURE 5:**
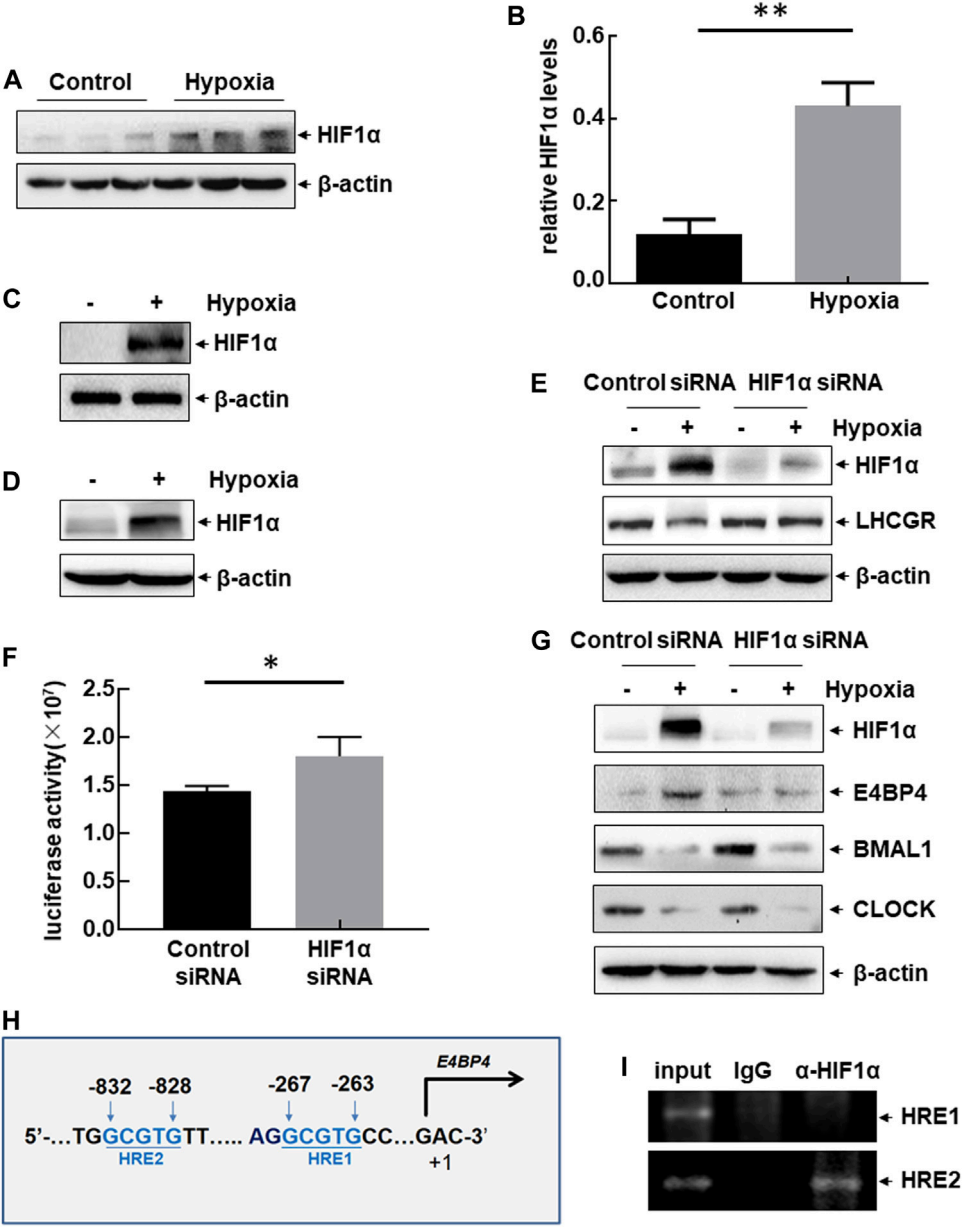
Hypoxia-induced dysregulation of clock-controlled LHCGR reduction in HIF1ɑ-dependent and ɑ-independent manners. **(A–B)** Female mice were treated as shown in Figure 1A. Ovaries were collected 48 h after hypoxic exposure, and the expression level of HIF1ɑ was detected. The quantitative results of HIF1ɑ expression (relative to β-actin) are shown **(B)** (*n* = 6, **p < 0.01). **(C–D)** Primary GCs **(C)** and KK1 cells **(D)** were cultured under normoxic or hypoxic conditions for 6 h. Then, the expression level of HIF1ɑ was detected. **(E)** Primary GCs were transfected with HIF1ɑ siRNA or control siRNA and then left untreated or subjected to hypoxic exposure. Cells were collected 6 h later, and the expression level of HIF1ɑ was detected. **(F)** KK1 cells were transfected with the LHCGR promoter-driven luciferase reporter in combination with siRNA targeting HIF1ɑ or control siRNA. The luciferase activity was detected 36 h after transfection ( *p < 0.05). **(G)** Primary GCs were transfected and treated as described for **(E)**, and the expression levels of circadian clock proteins were detected. **(H)** Potential HIF1ɑ-binding sites (HRE) were identified within mouse E4BP4 promoter regions. **(I)** ChIP assays were performed on the hypoxia-treated KK1 cells to detect the functional HRE within the mouse E4BP4 promoter region.

Hypoxia-inducible factor 1ɑ (HIF1ɑ) is a key signaling molecule that mediates various stress responses in multiple cells under hypoxic exposure (Kumar and Choi 2015). Therefore, we investigated whether HIF1ɑ is involved in the clock-controlled suppression of ovarian LHCGR expression in GCs. Upregulation of HIF1ɑ expression was observed in the ovaries of hypoxia-treated female mice (Figures 5A,B), and significant accumulation of HIF1ɑ was observed in hypoxia-treated primary GCs (Figure 5C) and KK1 cells (Figure 5D). HIF1ɑ siRNA was transfected into KK1 cells, followed by exposure to hypoxia to evaluate the role of HIF1ɑ in regulating LHCGR expression. Suppression of LHCGR expression was almost completely reversed by blocking HIF1ɑ induction under hypoxic exposure (Figure 5E). Moreover, knockdown of HIF1ɑ expression enhanced LHCGR promoter-driven luciferase activity in KK1 cells (Figure 5F). These results indicate that HIF1ɑ plays a negative role in regulating LHCGR expression in GCs.

Changes in the circadian clock protein levels were evaluated in the absence or presence of HIF1ɑ expression in hypoxia-treated KK1 cells. Blocking HIF1ɑ expression inhibited E4BP4 upregulation induced by hypoxic exposure but did not affect the decreased expression of CLOCK and BMAL1 under the same conditions (Figure 5G). These results indicate that dysregulation of circadian clock protein expression is induced by hypoxia through HIF1ɑ-dependent and independent pathways. We identified two HIF1-responsive elements (HREs) within the E4BP4 promoter region (Figure 5H). ChIP assay data further confirmed the recruitment of HIF1ɑ to the distal HRE (−832 to −828 bp) within the E4BP4 promoter after hypoxic exposure (Figure 5I). These results suggest that impaired expression of ovarian LHCGR mediated by the suppression of clock output signals (CLOCK and BMAL1) is not related to HIF1ɑ, but LHCGR reduction mediated by the activation of clock output signals (E4BP4) is controlled by HIF1ɑ.

### 3.6 LHCGR reduction resulted in impaired ovulation-related gene expression

**FIGURE 6:**
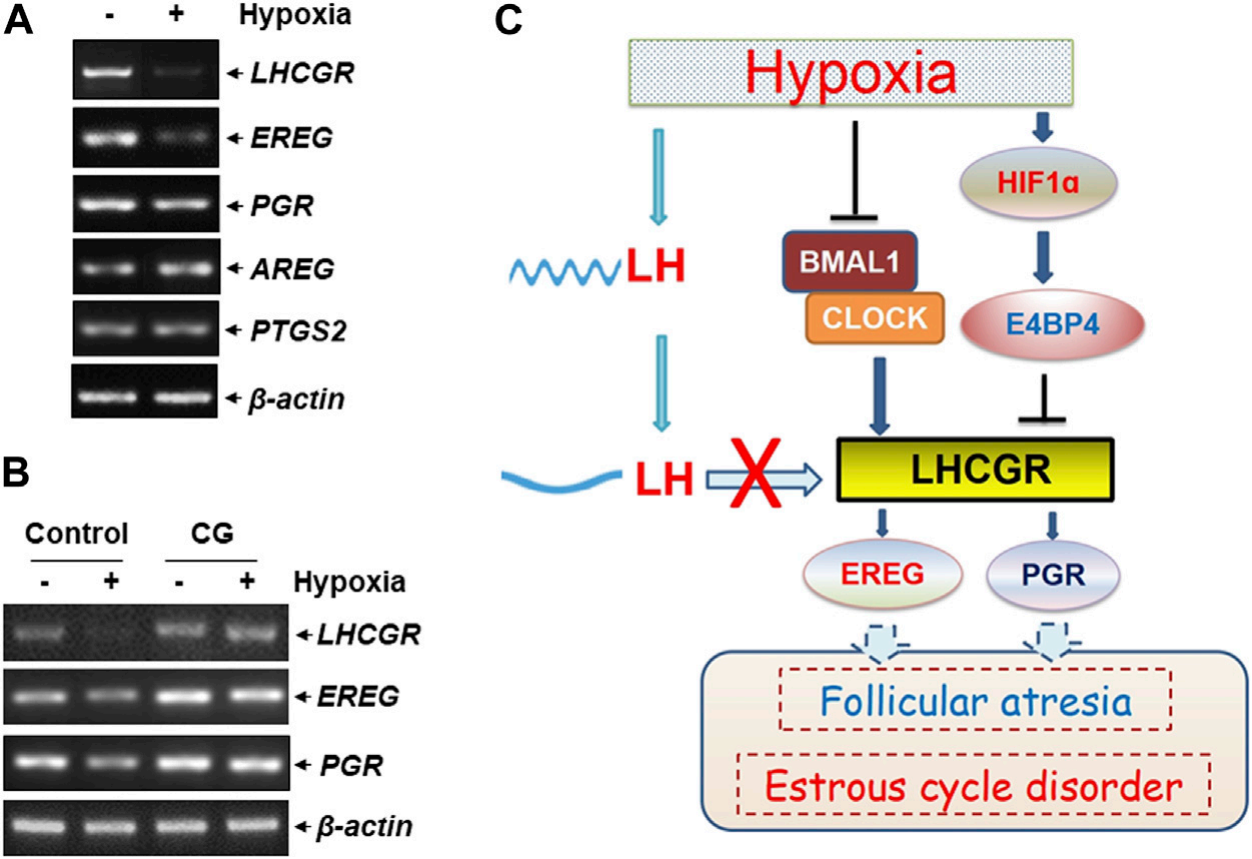
LHCGR agonist restored the impaired ovulation-related gene expression in the granulosa cells induced by hypoxia. **(A)** KK1 cells were untreated or exposed to hypoxia. The expression levels of LHCGR and the ovulation-related genes were detected. **(B)** KK1 cells were untreated or exposed to hypoxia with or without pretreatment of CG. The expression levels of LHCGR and the ovulation-related genes were detected. **(C)** Working model of circadian clock-controlled female reproductive dysfunction upon hypoxic exposure.

The functional outcome of suppressed LHCGR expression was observed in ovarian GCs under hypoxic exposure. The expression levels of several ovulation-related genes were analyzed in hypoxia-treated KK1 cells, including epiregulin (*EREG*), amphiregulin (*AREG*), progesterone receptor (*PGR*), and prostaglandin-endoperoxide synthase 2 (*PTGS2*). We found that along with the impairment of LHCGR expression, the levels of *EREG* and *PGR* expression decreased after hypoxic exposure (Figure 6A), and the response was efficiently recovered by pretreatment of GCs with the LHCGR agonist, chorionic gonadotropin (CG) (Figure 6B). Under the same conditions, the expression levels of other ovulation-related genes (*AREG* and *PTGS2*) did not change (Figures 6A,B). These results indicate that recovering LHCGR activation efficiently restores the impaired expression of ovulation-related genes in hypoxia-treated GCs.

LH surge is critical for maintaining ovulation and the estrous cycle (Troppmann et al., 2013; Mikhael et al., 2019). Based on the aforementioned results, we conclude that hypoxia exposure induces deregulation of circadian clock protein expression and the resultant LHCGR reduction in GCs in the ovary. This, combined with the decrease in rhythmic LH expression in the serum, results in GCs losing LH reactivity, leading to impaired ovulation-related gene expression, follicular atresia, and estrous cycle disorder in female mice under hypoxic exposure (Figure 6C).

## 4 Discussion

Female reproductive disorders occur at high altitudes; however, the mechanisms involved in harmful reproductive responses induced by hypoxia are unclear (West 2017; Shaw et al., 2018; Lorca et al., 2019). Sleep disorder frequently occurs as a stress response to the highlands, which results in disturbance of circadian clock protein expression and a variety of physiological abnormalities (Windsor and Rodway 2012; Bloch et al., 2015). Therefore, determining the impact of hypobaric hypoxia on circadian clock protein expression in the female reproductive system and the resulting HPO axis dysfunction may be helpful in finding novel avenues for ameliorating hypoxia-induced female reproductive disorders.

The contribution of the circadian clock to ovarian function is a focus point in reproductive medicine. Circadian clock genes have been shown to be expressed in all the functional cells in the ovary, including the GCs, TCs, oocytes, and interstitial or stromal cells. The rhythmic molecular clocks in these cells are critical for steroidogenesis, folliculogenesis, cellular differentiation, responsiveness to gonadotropins, and ovulation (Sellix 2015; Sen and Sellix 2016; Sen and Hoffmann 2020; Brzezinski et al., 2021; Shao et al., 2021). The LH surge is an important event in the control of ovulation. In nocturnal rodents, LH secretion increases in the afternoon and peaks 3–4 h into the night. The ovarian biological clock can regulate the synchronization of follicles with the LH surge and cause ovulation (Troppmann et al., 2013; Kobayashi et al., 2018; Mikhael et al., 2019). In this process, LHCGR expressed in the GCs and TCs contributes largely to mediating the time of ovulation due to the E-box located in the promoter region of LHCGR (Shimizu et al., 2011; Chen et al., 2013; Mereness et al., 2016; Wang et al., 2017; Wang et al., 2020). It has been reported that specific knockout or knockdown of BMAL1 expression in GCs or TCs decreases LHCGR levels and causes mice to lose sensitivity to LH stimulation (Shimizu et al., 2011; Chen et al., 2013; Mereness et al., 2016; Wang et al., 2017; Wang et al., 2020). Additionally, CLOCK mutant mice display an extended, irregular estrous cycle without an LH surge in the afternoon of proestrus (Miller et al., 2004). Knockdown of CLOCK expression also results in the impairment of LHCGR transcription in GCs (Shimizu et al., 2011). Therefore, CLOCK affects the possibility of ovulation and conception, mediated by the LH surge. Consistent with these reports, we found that the E-box within the mouse LHCGR promoter mediates CLOCK:BMAL1 heterodimer-controlled LHCGR transcription in GCs. Moreover, we also identified the D-box for the binding site of another circadian clock protein, E4BP4, within the promoter region of mouse LHCGR. However, in contrast to CLOCK and BMAL1, E4BP4 functions as a transcriptional suppressor of LHCGR in GCs. These results provide new evidence for circadian regulation of the LH surge and ovarian function in female mice.

The precise timing of rhythmic events in the reproductive tract is synchronized with the light/dark (L/D) cycle (Sen and Sellix 2016; Brzezinski et al., 2021). In the current study, we demonstrated that the expression of ovarian circadian clock proteins (CLOCK, BMAL1, and E4BP4) could also be entrained by hypoxic exposure. At the molecular level, the capacity of circadian clocks to exhibit flexibility under hypoxic exposure depends on the structural similarity between the circadian clock activators (CLOCK and BMAL1) and HIF1 subunits (HIF1α and HIFβ), all of which belong to the basic helix–loop–helix (bHLH)–Per–Arnt–Sim (PAS) family. PAS domains within these molecules function as environmental response modules and mediate interactions between HIF1 subunits and circadian activators. Therefore, the HIF1–CLOCK/BMAL1 interaction may integrate hypoxic changes into circadian transcriptional programs in certain organs or tissues (Wu and Rastinejad 2017). In GCs, although we observed the induced interaction of HIF1α with CLOCK and BMAL1 after hypoxic exposure (data not shown), blocking HIF1α accumulation did not affect the decrease in expression of both clock activators. Thus, these results exclude the possibility that the reduction of CLOCK:BMAL1 activity entrained by hypoxic exposure is a downstream event following HIF1α induction in the ovary. Under the same hypoxic conditions, upregulation of E4BP4 was controlled by HIF1α in GCs, indicating a new functional link between hypoxia signaling and circadian clock proteins in the female reproductive system.

The CLOCK:BMAL1 heterodimer plays a broad role in regulating the expression of genes controlling ovarian function under physiological conditions, such as *LHCGR*, *STAR* (rate-limiting enzymes for P_4_ synthesis), *CYP11A1*, and *CYP19A1* (rate-limiting enzymes for E_2_ synthesis) (Sellix 2015; Sen and Sellix 2016; Sen and Hoffmann 2020; Brzezinski et al., 2021; Shao et al., 2021). However, hypoxia-induced CLOCK and BMAL1 reduction resulted in a decrease in LHCGR levels and did not affect the expression of other CLOCK:BMAL1 transcriptional targets (*STAR*, *CYP11A1*, and *CYP19A1*) in the ovary (data not shown). The selective transcription of CLOCK:BMAL1 downstream target genes in the ovary is determined by a hypoxic signal, but in a currently unidentified HIF1α-independent manner. Moreover, E4BP4 has been shown to function as a transcriptional suppressor of PTSG2, the rate-limiting enzyme for prostaglandin synthesis (Li et al., 2011). However, according to our results, the levels of PTSG2 did not change in GCs after hypoxic exposure. Therefore, the binding selectivity of E4BP4 to the LHCGR promoter instead of the PTSG2 promoter appears to be determined by HIF1α in hypoxia-treated GCs.

Another concern is that LH has been shown to entrain the majority of clock genes (*BMAL1*, *PER2*, *REV-ERBα*, and *DBP*) with robust rhythmic expression in mature GCs (Kobayashi et al., 2018). Therefore, in addition to mediating the suppression of LHCGR transcription, a decrease in BMAL1 expression may also be a downstream signaling event following the impairment of LHCGR responsiveness. If this is the case, the positive feedback regulation between LHCGR and BMAL1 will expand the ovarian disorder induced by hypoxia.

In conclusion, we revealed a new mechanism involving circadian clock protein-controlled ovarian disorders under hypoxic exposure. CLOCK, BMAL1, and E4BP4 can be entrained by hypoxic signaling, which synergistically promotes LHCGR reduction in GCs and results in ovarian dysfunction. Targeting circadian clock proteins or recovering the sensitivity of GCs to LH surge might be helpful in overcoming female reproductive disorders occurring in the highlands.

## Data Availability

The raw data supporting the conclusion of this article will be made available by the authors, without undue reservation.
